# Supplementary material for: Expression of Tryptophan 2,3-Dioxygenase in Metastatic Uveal Melanoma
Source: Cancers (Basel). 2020 Feb 10;12(2):405. doi: 10.3390/cancers12020405 (PMC7072257; doi:10.3390/cancers12020405)
Supplement: Supplementary file 1 [file cancers-12-00405-s001.pdf]

Supplementary Files

Expression of tryptophan 2,3-dioxygenase in metastatic uveal melanoma

Mizue Terai, Eric Londin, Ankit Rochani, Emma Link, Bao Lam, Gagan Kaushal, Alok Bhushan, Marlana Orloff, and Takami Sato

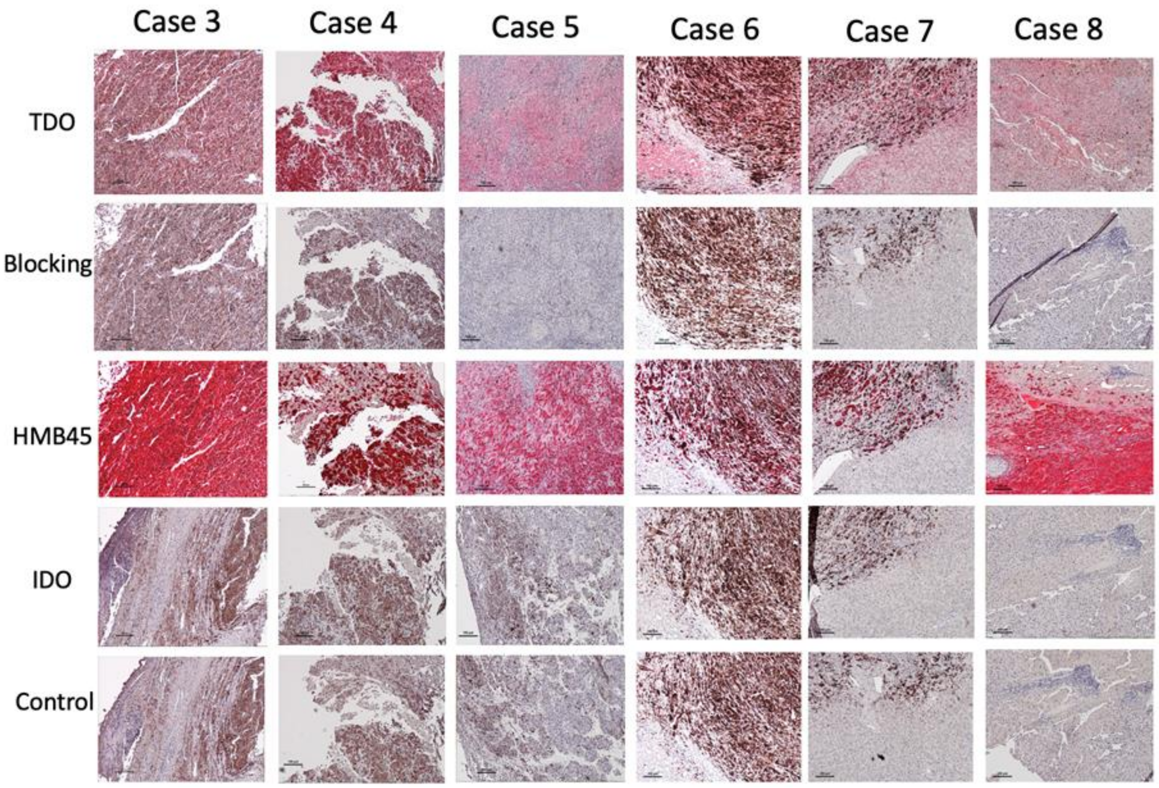

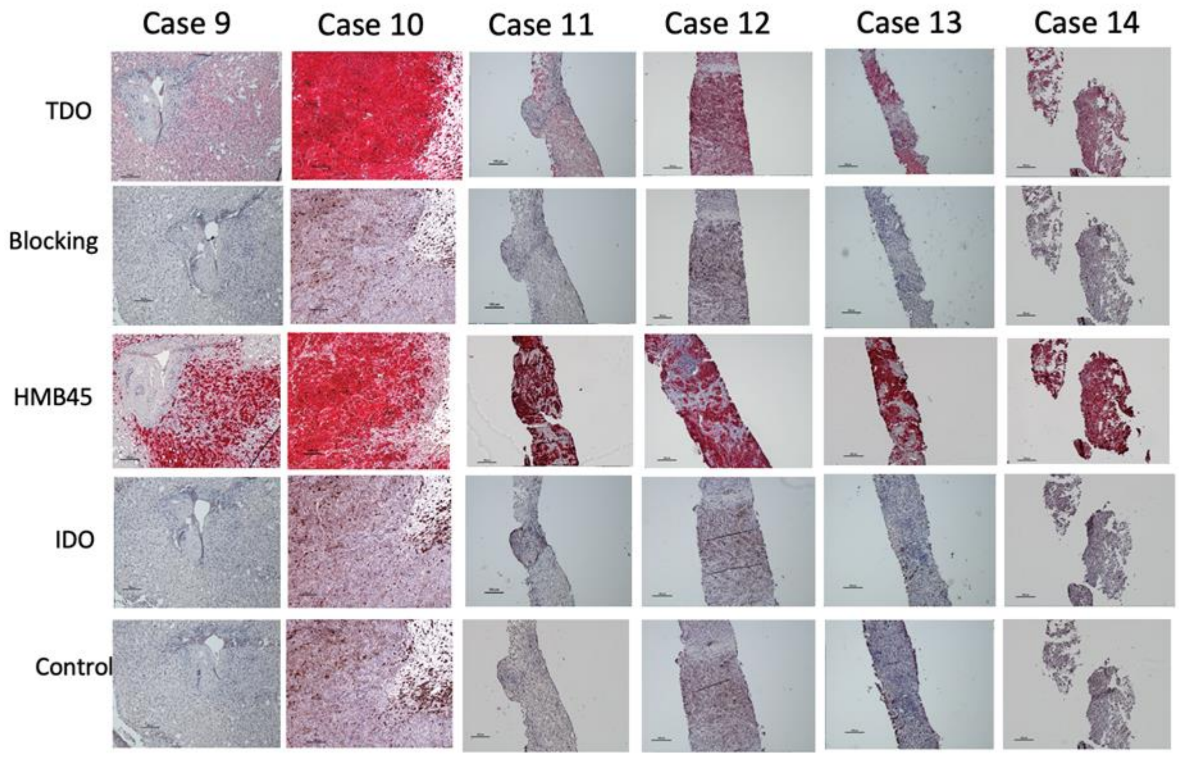

Figure S1. Immunohistochemical (IHC) staining of 12 liver metastasis specimens. Blocking: TDO antibody was blocked with blocking peptide. IgG Control: Isotype-matched IgG. Magnification: x100. Scale bar=100 μm.

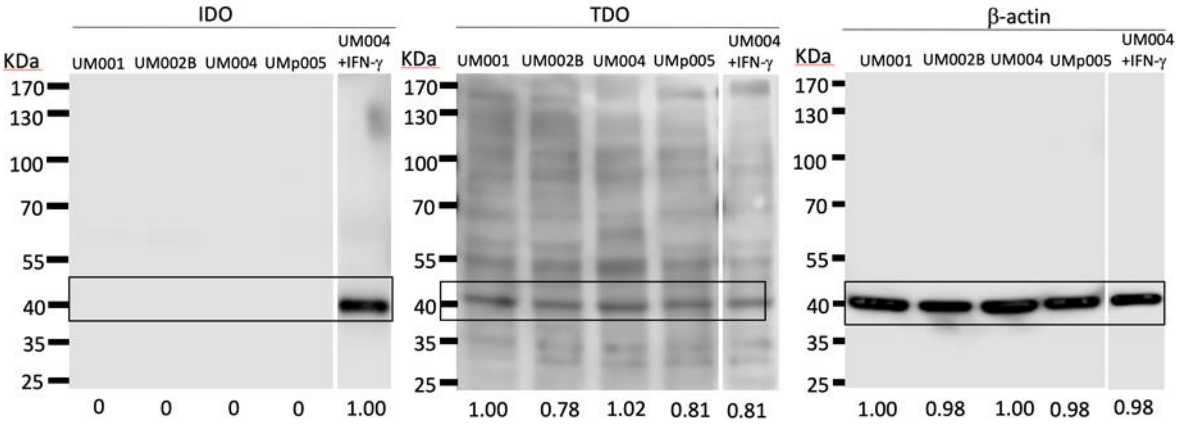

Figure S2. Whole blots for western blot shown in Figure 3B.

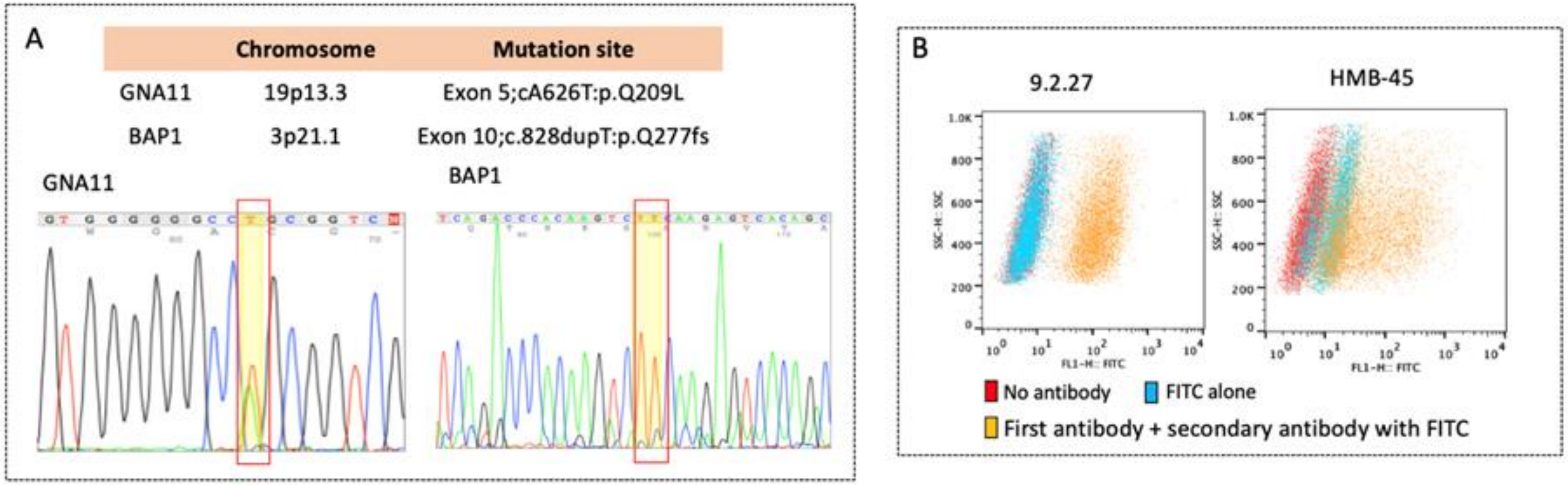

Figure S3. Characterization of UMp005 cell line. (**A**) Sanger sequence mutations analysis. (**B**) Detection of melanoma markers, HMW-MAA (9.2.27) and HMB45.

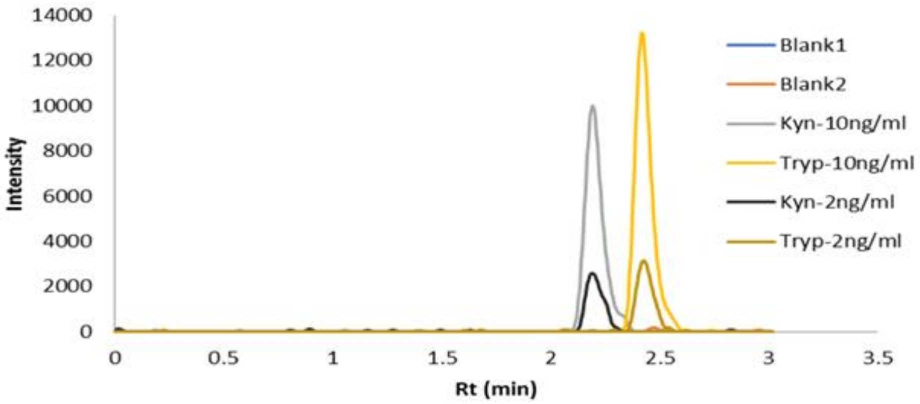

Figure S4. Representative XIC for Kyu (m/z: 209.09-209.1) and Trp (m/z: 205.09-205.1). It shows LLOD and LLOQ concentrations as 2 ng/ml and 10 ng/ml for both kynurenine and tryptophan, respectively are shown for the quantitation method.

Table S1. Summary of clinicopathological phenotypes of 80 patients with primary uveal melanoma in TCGA Database.

Clinicopathological Phenotype	Number (*n*)
Gender	Male *n* =4 5<br>Female *n* = 35
Chromosome 3 copy number	Monosomy 3 *n* = 37<br>Disomy 3 *n* = 37<br>Unknown gene analysis *n* = 6
BAP1 Mutation	BAP1 Mutation *n* = 35<br>BAP1 Wild type *n* = 45
TCGA SCNA cluster	Cluster 1 *n* = 15<br>Cluster 2 *n* = 23<br>Cluster 3 *n* = 22<br>Cluster 4 *n* = 20
Development of metastatic disease	Metastatic *n* = 25<br>No development of metastatic *n* = 54<br>Unknown *n* = 1

Table S2. List of abbreviations in figure 6A.

No.	Abbreviation	Full Form
1	UVM	Uveal Melanoma
2	UCS	Urothelial Carcinomas
3	UCEC	Uterine Corpus Endometrial Carcinoma
4	THYM	Thymoma
5	THCA	Thyroid carcinoma
6	TGCT	Testicular Germ Cell Tumors
7	STAD	Stomach adenocarcinoma
8	SKCM	Skin Cutaneous Melanoma
9	SARC	Sarcoma
10	READ	Rectum adenocarcinoma
11	PRAD	Prostate adenocarcinoma
12	PCPG	Pheochromocytoma and Paraganglioma
13	PAAD	Pancreatic adenocarcinoma
14	OV	Ovarian serous cystadenocarcinoma
15	MESO	Mesothelioma
16	LUSC	Lund squamous cell carcinoma
17	LUAD	Lung adenocarcinoma
18	LIHC	Liver hepatocellular carcinoma
19	LGG	Brain Lower Grade Glioma
20	LAML	Acute Myeloid Leukemia
21	KIRP	Kidney renal papillary cell carcinoma
22	KIRC	Kidney renal clear cell carcinoma
23	KICH	Kidney Chromophobe
24	HNSC	Head and Neck squamous cell carcinoma
25	GBM	Glioblastoma multiforme
26	ESCA	Esophageal carcinoma
27	DLBC	Diffuse large B-cell lymphoma
28	COAD	Colon adenocarcinoma
29	CHOL	Cholangiocarcinoma
30	CESC	Cervical squamous cell carcinoma and endocervical adenocarcinoma
31	BRCA	Breast invasive carcinoma
32	BLCA	Urothelial Bladder Carcinoma
33	ACC	Adrenocortical Carcinoma

Table S3. Primer sequence of TDO2, IDO and GAPDH.

RNA	Primer Sequence for RT-PCR
TDO2 sense	5′-TTGGACTTCAATGACTTCAGAGA-3′
TDO2 antisense	5′-TGCCCAGCATTCTTGTGC-3′
IDO1 sense	5′-GAGGAGCAGACTACAAGAATG-3′
IDO1 antisense	5′- GCATACAGATGTCTCTGCTATG-3′
GAPDH sense	5′-ACCACAGTCCATGCCATCAC-3′
GAPDH antisense	5′-TCCACCACCCTGTTGCTGTA-3′
**RNA**	**Primer Sequence for Quantitative PCR**
TDO2 sense	5′-ACTCCCCGTAGAAGGCAGCGAA-3′
TDO2 antisense	5′-CGGTGCATCCGAGAAACAACCT-3′
IDO1 sense	5′-GGGGCACCAGAGGAGCAGACT-3′
IDO1 antisense	5′- AGACGTGCAAGGCGCTCTGAC-3′
GAPDH sense	5′-GAGTCAACGGATTTGGTCGT-3′
GAPDH antisense	5′-TTGATTTTGGAGGGATCTCG-3′

Table S4. %CV and % recovery for Kyn linearity concentration (2–2000 ng/mL) range for inter and intra-day runs.

Concentration (ng/mL)	% CV		% Recovery	
	Intra Day	Inter Day	Intra Day	Inter Day
2	2.73	1.12	126.19	127.20
10	1.87	1.09	109.75	108.91
40	3.24	0.69	85.94	86.37
100	1.83	0.36	80.62	80.82
500	1.81	3.14	94.27	92.22
1000	2.23	0.68	98.02	97.55
2000	1.23	2.17	102.72	101.17

© 2020 by the authors. Licensee MDPI, Basel, Switzerland. This article is an open access article distributed under the terms and conditions of the Creative Commons Attribution (CC BY) license (http://creativecommons.org/licenses/by/4.0/).
